# Optical-Waveguide Based Tactile Sensing for Surgical Instruments of Minimally Invasive Surgery

**DOI:** 10.3389/frobt.2021.773166

**Published:** 2022-01-19

**Authors:** Yue Li, Jian Hu, Danqian Cao, Stephen Wang, Prokar Dasgupta, Hongbin Liu

**Affiliations:** ^1^ School of Biomedical Engineering & Imaging Sciences, Faculty of Life Sciences and Medicine, King’s College London, London, United Kingdom; ^2^ Huawei Technologies R&D UK Ltd., Cambridge, United Kingdom; ^3^ Guy’s Hospital, Faculty of Life Sciences and Medicine, King’s College London, King’s Health Partners, London, United Kingdom

**Keywords:** tactile sensor, minimally invasive surgical, 3D surface, optical sensor, multi-point force measurement

## Abstract

In recent years, with the rapid development of minimally invasive surgery (MIS), the lack of force sensing associated with the surgical instrument used in MIS has been increasingly a desirable technology amongst clinicians. However, it is still an open technical challenge to date since most existing tactile sensing principles are not suitable to small 3-dimensional (3D) curved surfaces often seen in surgical instruments, and as a result multi-point force detection cannot be realized. In this paper, a novel optical waveguide-based sensor was proposed to deal with the above research gap. A sensor prototype for curved surfaces resembling the surface of dissection forceps was developed and experimentally evaluated. The static parameters and dynamic response characteristics of the sensor were measured. Results show that the static hysteresis error is less than 3%, the resolution is 0.026 N, and the repeatability is less than 1.5%. Under a frequency of 12.5 Hz, the sensor could quickly measure the variation of the force signal. We demonstrated that this small and high-precision sensitive sensor design is promising to be used for creating multiple-point tactile sensing for minimally invasive surgical instruments with 3D surfaces.

## Introduction

MIS is a surgical technique that is performed through keyholes. MIS has the advantages of less trauma, less pain, and rapid recovery (Puangmali et al., 2008). However, one of the main limitations in MIS is the lack of tactile force feedback because surgeons need to operate instruments through small incisions (Okamura, 2018). The loss of tactile information will lead to several problems: the characteristics of organs or tissues cannot be evaluated by manual palpation, which brings some difficulties to disease diagnosis. Also, an excessive amount of pressure could lead to hemorrhage or perforation during the surgery, which may cause fatal threats for patients (Zhou et al., 2016). Finally, the surgical instrument may slip and damage the tissue when grasping or suturing tissue due to insufficient or excessive force. Therefore, a tactile sensor, which can detect the contact force along the surgical instruments, would be essential to reduce such risks and thus improve safety accordingly.

Recently, in order to solve the problem of insufficient force-sensing ability of MIS, considerable progress has been made on the development of tactile sensing technologies. Some commonly researched force transduction techniques are based on displacement, current, pressure, resistive, capacitive, piezoelectric, and optical methods. Overall, two types of sensing have been widely studied: indirect force sensing and direct force sensing (Yu et al., 2018). For indirect force sensing, the measuring elements are usually arrayed on the joint actuation unit, instrument shaft, and articulated joint (Puangmali et al., 2008). (Jolaei et al., 2019) developed a particle-swarm optimization method based on the displacement to calculate the contact force between radio-frequency ablation (RFA) catheter and atrial tissue, and the accuracy of the module was 91.5%. However, the friction at joints and backlash in drive mechanisms could lead to measurement error in this process (Zhao and Nelson, 2015). detected the grip force by detecting the current of the motor in the driving joint. Although it was a simple method of measuring force, the result was bigger than the actual force applied to the instrument due to the friction effect and inertia at the connecting rod joints (Miyazaki et al., 2015). estimated the forceps contact forces by the differential pressure of the pneumatic cylinders and determined contact force values with a resolution of as good as 0.25 N. Still, the characteristics including nonlinearity, system uncertainty, and joint interference led to inconsistent and unreliable measurement results. (Haraguchi et al., 2015) introduced a three-degree-of-freedom (DOF) continuum model considering expansion and contraction of the flexible joint, which allowed three-axis force sensing on the forceps tip with 0.37 N estimation error. (Roshan et al., 2019; Dalvand et al., 2014; Trejos et al., 2017). placed the strain gauge on the instrument axis of the abdominal cavity to obtain the perception of touch and force. However, for the electrical-based force sensors, the wiring complexity constrains the miniaturization. (Wee et al., 2016) developed a two-degree freedom force-sensing sleeve using a full bridge strain gauge as a sensing element, and the force was measured up to 10 N. But the low sensitivity and hysteresis limited its use (Haghighipanah et al., 2017). estimated the external forces acting on the robot in all four quadrants using cable stretch and dynamics-based methods utilizing system dynamics and unscented Kalman filter (UKF). Although these indirect measurement methods can realize the force sensing of surgical instruments, the measurement accuracy is seriously limited due to the model error, estimation deviation, signal interference, and transmission loss.

In order to directly measure the force acting on the tip of the surgical instrument, researchers tried to place the force sensors on the gripper jaw, where would not be affected by friction and other interference forces. (Hou et al., 2021) developed a 2 mm × 2 mm sensor based on the piezoresistive principle for force sensing of grasping the head and realized detection resolution 0.1 N, but crosstalk still existed in each direction, and the characteristics of sensors were affected by temperature. (Kim et al., 2015) presented a novel force sensor based on the capacitive transduction principle and successfully provided two-DOF force information, but the miniaturization, assembly and sterilization were not considered. For electrical-based force sensors, challenges include complex packaging and electromagnetic interference (Akinyemi et al., 2021). As a result, many researchers have applied optical fiber sensors to surgical devices. (Lim et al., 2014) and (He et al., 2014) applied optical FBGs on the surgical grasper to measure the grasping force and the resolution of the grasping force reached 11 mN. But the measurement system needed a temperature compensation grating to alleviate the thermal drift of axial force sensing.

Overall, most of these sensors used for MIS are only applicable for 2D planes and none of the existing work offers solutions on the pressure measurement for a 3-dimensional (3D) curved surface, such as that of curved dissecting forceps. In addition, the space limitation of the jaw puts forward very high requirements for the size of the sensing element and the existing solutions only enable sensing with very limited spatial resolution, which is not sufficient for detecting multiple contacts between the instruments. To advance the Frontier of this research, a 3D optical-based tactile sensing array used for detecting the contact force in MIS is presented in this paper and the characteristics of hysteresis, resolution, repeatability and dynamic response are studied. Furthermore, combined with the properties of MIS, the application feasibility of this sensor is discussed.

## Sensor Concepts

### Working Principle

**FIGURE 1:**
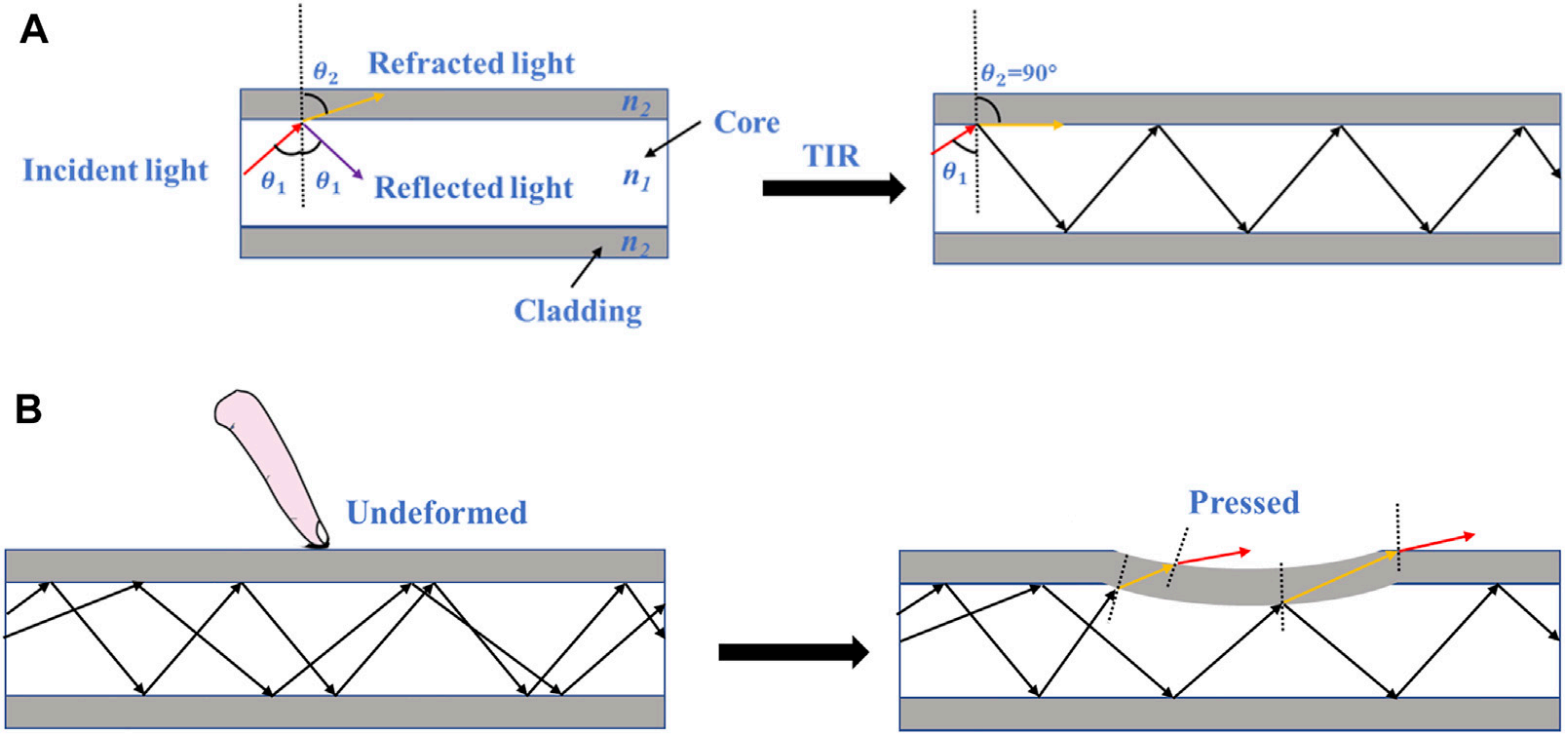
Working principle of optical fiber. **(A)** Total internal reflection occurs when the light is completely reflected when they arrive at the boundary between core and cladding, **(B)** Ray diagrams of the optical fiber when it is undeformed and pressed.

We developed a tactile array sensor based on optical waveguide principle because it has unique advantages of compact size, inherent electrical safety, and immunity to electromagnetic interferences. Optical fiber is composed of core and cladding, and its working principle is shown in Figure 1A. $n_{1}$ is the refractive index of the core and $n_{2}$ is the refractive index of the cladding. According to (Caucheteur et al., 2015), the light will totally internally reflect (TIR) in the interface of material with different refractive indexes when met the following conditions:

a. Light transmits from a denser medium into a less dense medium. ( $n_{1}$ > $n_{2}$ )
b. The angle of incidence $\left(\theta_{1}\right)$ must be greater than the critical angle (ϕ) .

Based on refraction’s law, Eq. 1 is established:

$$n_{1}\sin\left(\theta_{1}\right)=n_{2}\sin\left(\theta_{2}\right)$$ (1)

where $\theta_{1}$ is the incidence angle, $\theta_{2}$ is the refraction angle. When $\theta_{2}=90{}^{\circ},$ the light will be totally reflected and then the critical angle ϕ could be calculated by Eq. 2:

$$\phi =\theta_{1}=\arcsin\left(\frac{n_{2}}{n_{1}}\right)$$ (2)

When the optical fiber is pressed, TIR is no longer satisfied. As shown in Figure 1B, part of the light would escape through the core and pass through the cladding due to the deformation, leading to light loss. Therefore, the force can be obtained by measuring the change of light intensity.

### Sensor Design

According to the sensing principle, a polyurethane elastomer VytaFlex 20 (Smooth-On Inc.) with the refractive index 1.47 and a silicone elastomer Ecoflex 00-50 (Smooth-On Inc.) with the refractive index 1.40 were selected as the core and cladding, respectively. Thus, the critical angle is 72.2°. Table 1 summarizes the main characteristics of VytaFlex 20 and Ecoflex 00-50 (Bai et al., 2020). It is clear that the materials are stretchable, elastic, and highly transparent.

**TABLE 1:** Properties of Ecoflex 00-50 and VytaFlex 20.

Material	Product	Elongation at break (%)	Tensile strength (MPa)	100% modulus (kPa)	Color	Refractive index
Silicone Elastomer	Ecoflex 00-50	980	2.3	83	Translucent	1.40
Polyurethane Elastomer	VytaFlex 20	1,000	1.4	340	Clear	1.47
					Ember	

**FIGURE 2:**
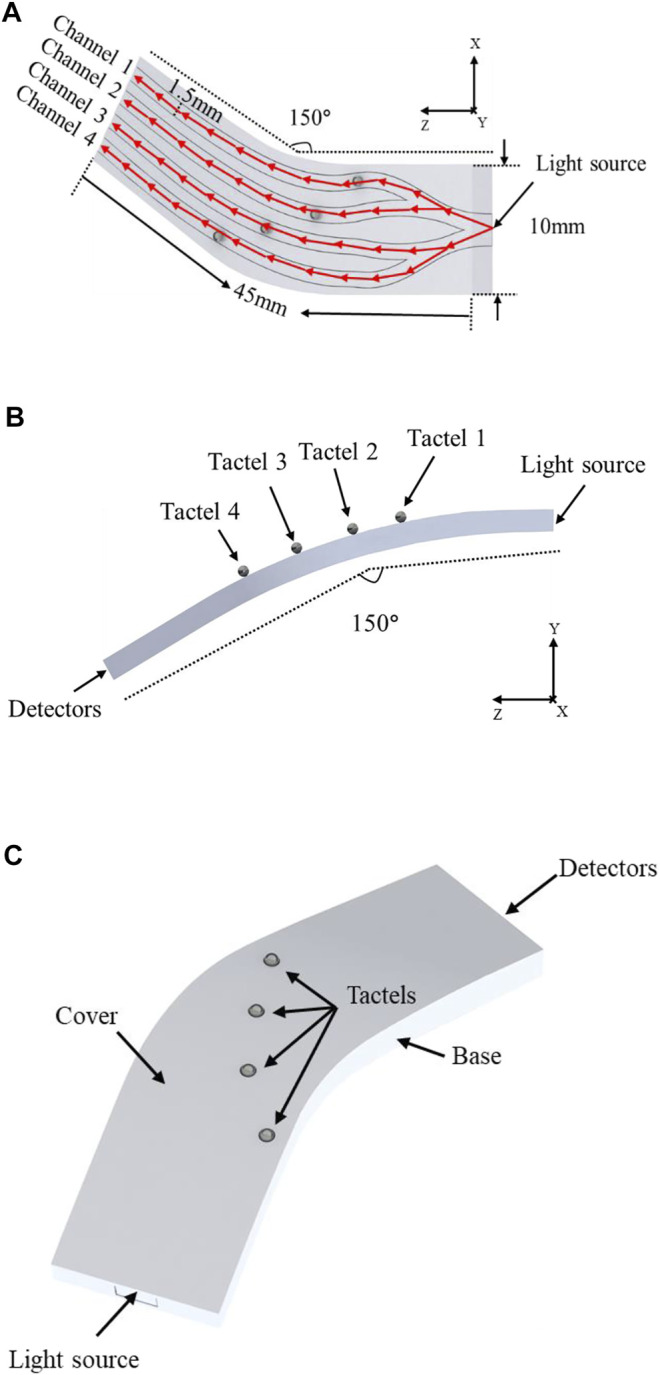
The layout of the proposed multi-point sensor. **(A)** The optical path of the waveguide, **(B)** the diameter of bending and the positions of tactels on the 3D surface, **(C)** Encapsulated sensor.

A 3D multi-point tactile sensor was designed, and the layouts of the sensor are shown in Figure 2. The shape of the sensor was designed to fit the surface of curved dissecting forceps with a width of 10 mm and a bending angle of 30°. Parameters of sensors could be adjusted according to the different detecting forceps. In order to apply this sensor on a complex 3D surface and detect as many multiple contacts as possible between the forceps, four tactile sensing elements were designed while the input waveguide was bifurcated into four independent channels. The channel is a 45 mm long curved rectangular fiber with a cross-section of 1.5 mm (height) by 1.5 mm (width) and the interval between channels is 1 mm to ensure each element is independent of the other. Meanwhile, steel balls with a diameter of 0.75 mm were selected as the tactel units to avoid any sharp contacts in MIS and they were fixed to the corresponding positions in the sensor. Therefore, when force is applied on the sensing point, distortion of the waveguide would result in light intensity loss. Accordingly, by detecting the light intensity signal, the value and position of the force from the tactile sensor can be obtained in real-time.

### Simulation

**FIGURE 3:**
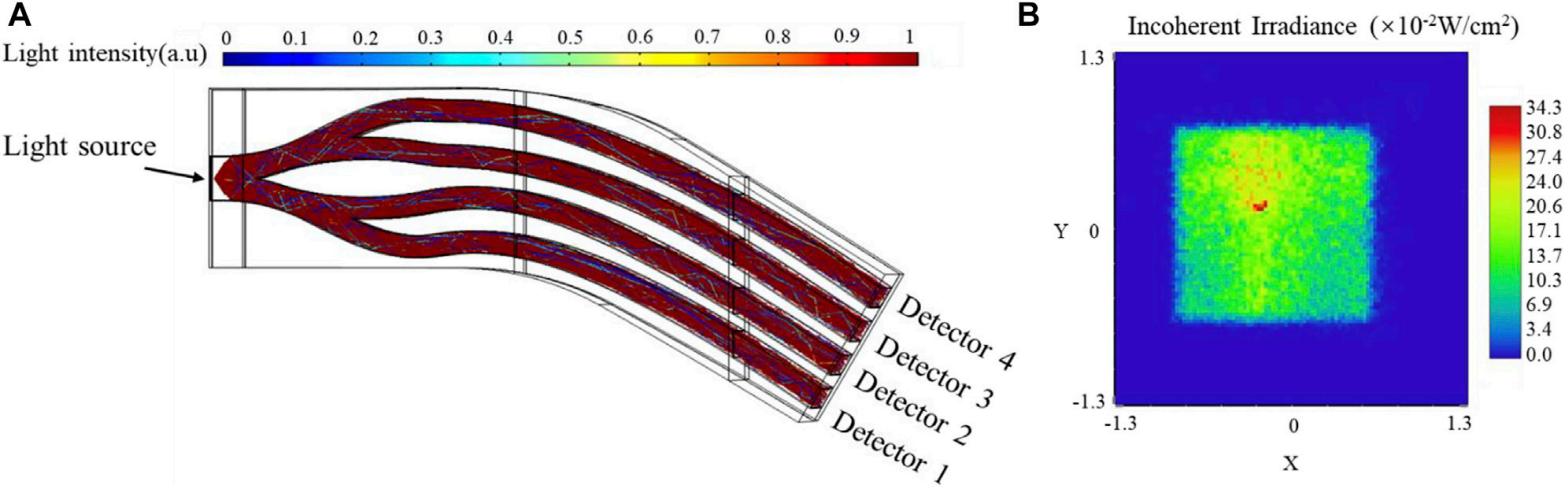
Simulation result of the undeformed sensor **(A)** Ray tracing, **(B)** The inherent radiation distribution map of channel 1.

The light utilization of the sensor is calculated with COMSOL Multiphysics to verify the feasibility of the design. COMSOL Multiphysics is a cross-platform finite element analysis and multiphysics simulation software, which allows coupled optical and mechanical simulation. In this paper, the mechanical simulation results were used as the initial dependent variable of the optical simulation in COMSOL and finally figured out the relationship between the output intensity of the sensor and the pressure. The simulation parameters of materials were set according to Table 1. The output of each channel was set as a detector and the deposited ray power was calculated. Total source power was 1 W and emitted with a cone angle of 60°. Figure 3 shows the ray tracing of the undeformed sensor and the inherent radiation distribution map of channel 1. The simulation results demonstrate that the initial intensity of four channels can reach 21.87, 17.21, 16.82, and 19.11%, respectively, where the total utilization of light is 75.02%.

**FIGURE 4:**
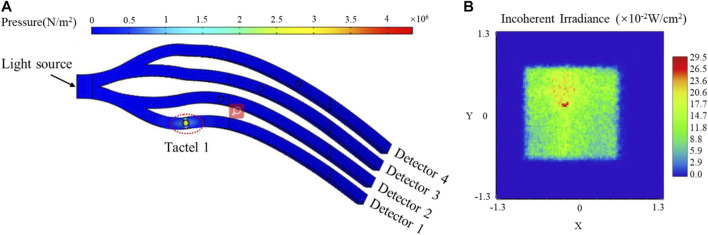
The simulation results of channel 1 with 1,500 kPa applied. **(A)** Deformation, **(B)** The inherent radiation distribution map.

Figure 4 shows the deformation and inherent radiation distribution of channel 1 when 1,500 kPa was applied at tactel 1. Accordingly, the maximum inherent radiation decreased from 34.3 × 10^−2^ W/cm^2^ to 29.5 × 10^−2^ W/cm^2^, while the output intensity of channel 1 decreased from 21.87 to 17.94%. It can be explained that the TIR is destroyed remarkably due to the deformation and part of light changes the original path and escapes from channel 1.

**FIGURE 5:**
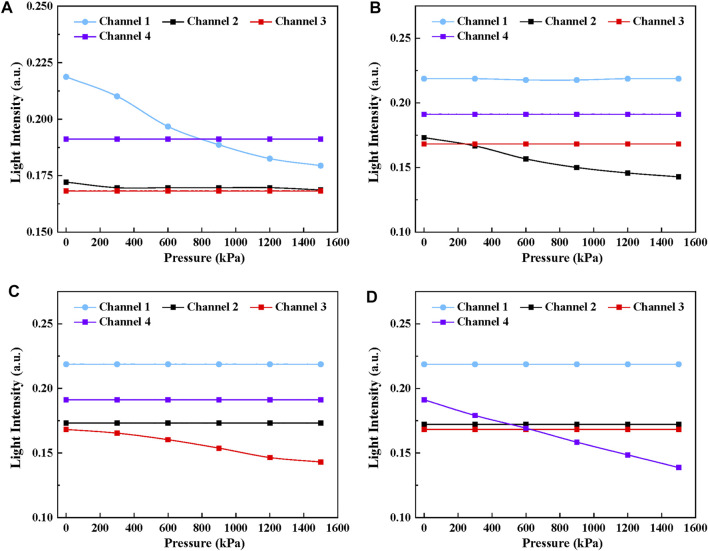
The simulation results of the tactile sensor with the pressure ranging from 0 to 1,500 kPa applied. **(A)**Channel 1, **(B)** Channel 2, **(C)** Channel 3, **(D)** Channel 4

Figure 5 demonstrates the simulation results when the pressure ranging from 0 to 1,500 kPa was applied on tactel 1 to tactel 4 of the proposed sensor. Significantly, the intensity of the deformed channel decreases linearly with the growth of the pressure while the signal intensity of other channels remains unchanged. Therefore, it can be concluded that the tactile sensor design can guarantee the sensing unit’s independence and no signal crosstalk occurs during experiments, which is essential for locating the force position. The simulation results provide theoretical guidance and support for the experiment.

### Fabrication Process

**FIGURE 6:**
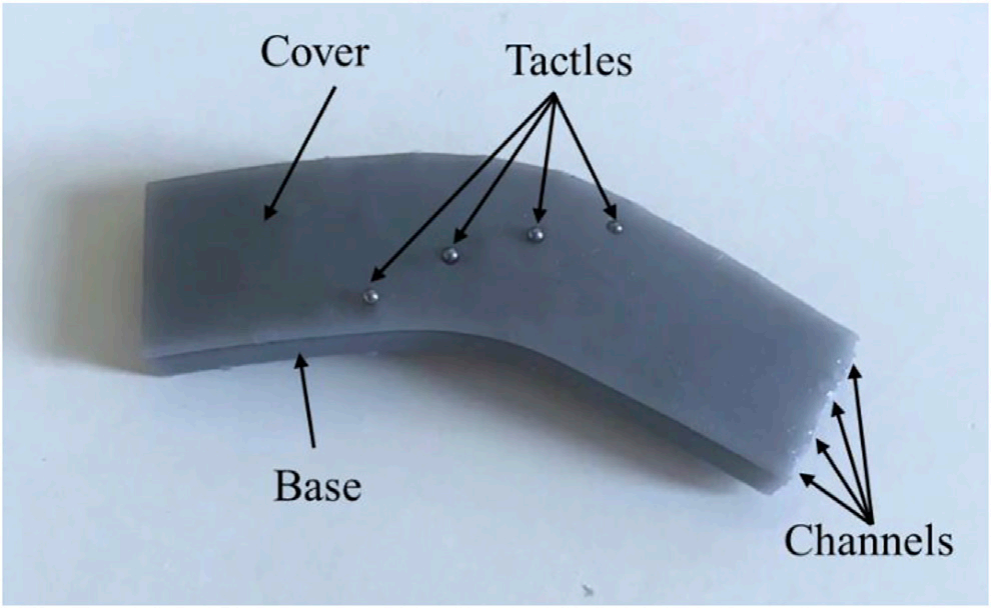
Encapsulated sensor for a 3D surface.

We fabricated the tactile sensor by replica molding. The fabrication process to embed the optical fiber was as follows: (a) First of all, the structure of the sensor was designed with Solidworks and printed by a 3D printer. (b) Then the 3D-printed mold was first left in the oven at 50°C for 1 h to remove residuals that may affect the curing of the material. Then the surface was cleaned and wiped with alcohol. (c) After, Ecoflex 00-50 Part A and B were mixed at a 1:1 ratio with a stir stick for 3 min. (d) After that, the mixed solution was put into the vacuum pump chamber to remove bubbles. The cladding layer was prepared by spin coating Ecoflex 00–50 to ensure the coating was even and uniform. The thickness of the cladding was 0.2 mm and the cladding cured at room temperature for 4 h. (e) However, the liquid can easily flow out due to gravity when pouring the core solution because the input and output sections were not on the same level. So a matched cover was proposed to seal the exposed channels tightly and the wax was selected to seal the outputs for preventing the fluid from flowing. (f) Then VytaFlex 20 Part A and B were mixed at a 1:1 ratio for 3 min. (g) The solution was injected into the channel base with a syringe and cured at room temperature for 16 h to form the core of the waveguide. (h) Removed the cover and repeated (c) and (d) to make the upper cladding. (I) Finally, the steel balls were placed on the expected positions of each channel and a silicone elastomer Ecoflex 00-50 (Smooth-On Inc.) was used to glue the press buttons with a rigid cover (designed to protect soft channels from external light and fix the press buttons). Epoxy (Thorlabs Inc. G14250) was used to glue the sensor mold with the cover. Therefore, a tactile sensor suitable for a 3D surface with four sensing units was obtained, as shown in Figure 6.

## Experiments and Results

### Experimental Platform

**FIGURE 7:**
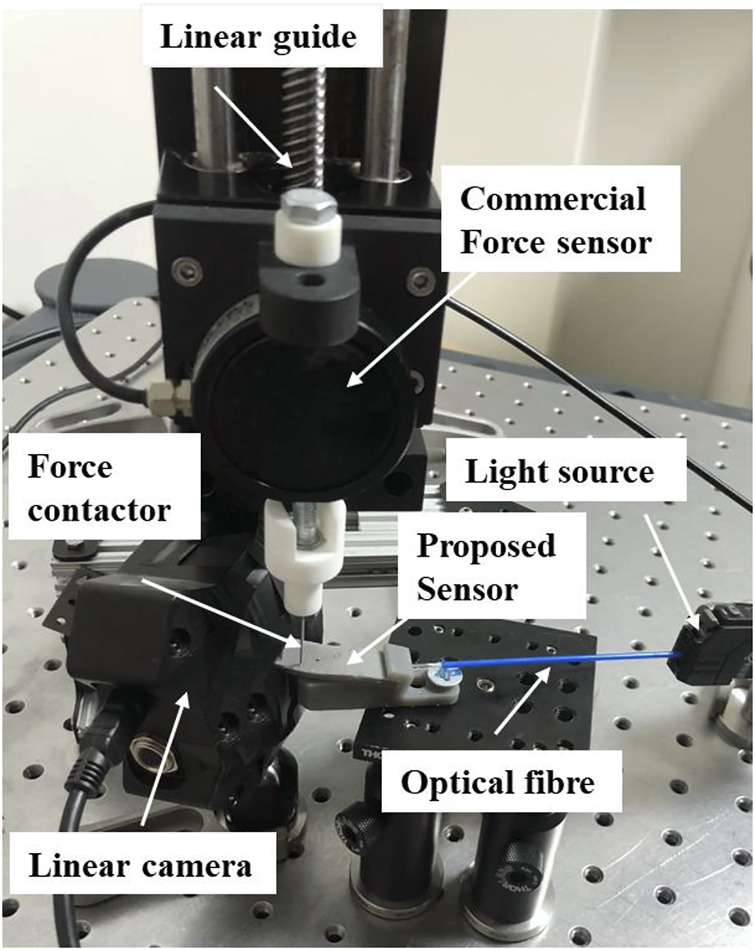
The experimental setup consists of a light source, a force contactor, the proposed sensors, a motorized linear guide, a load cell force sensor and a linear camera.

To characterize the high performance of our flexible sensor, a series of experiments were conducted. The experimental setup is shown in Figure 7. The platform consists of a light source (Keyence Ltd. FS-N11MN), a force contactor, the proposed sensors, a motorized linear guide (Newmark Systems Inc. ET-200-21), a load cell force sensor (Richmond Industries Ltd. ‘S' Beam Load Cells of 900 Series) and a linear camera (Thorlabs Ltd. LC100, 350–1100nm, 2048 Pixel Linear Si CCD Array). The laser emits the light and is transmitted to the input of the sensor, then Arduino is used to control the movement of the linear guide so that the force contactor can apply a force to the sensing unit. The magnitude of the force can be measured by the load cell force sensor. As previously explained, normal forces applied on the sensor causes a deformation of the waveguide, resulting in a light intensity decrease at the output section. Finally, the linear camera is used to detect the output signal and it can be displayed on the computer in real-time.

### Experiment Result

In order to demonstrate the feasibility of the above design of the tactile sensor, a series of experiments were carried out and the parameters such as the hysteresis, resolution, repeatability, and dynamic response were analyzed.

Before each experiment, the system was calibrated by adjusting the incident angle to 60° and fixing the position of the sensor to ensure equal initial output light intensity. When testing the sensor characteristics, the pressure was applied to the tactel of the sensor and gradually increased at a rate of 300 kPa. The peak value of the light intensity was recorded and the operation was repeated more than five times at each tactel ranging from 0 to 1,500 kPa. Finally, the output intensity under different forces was obtained by averaging the data.

**FIGURE 8:**
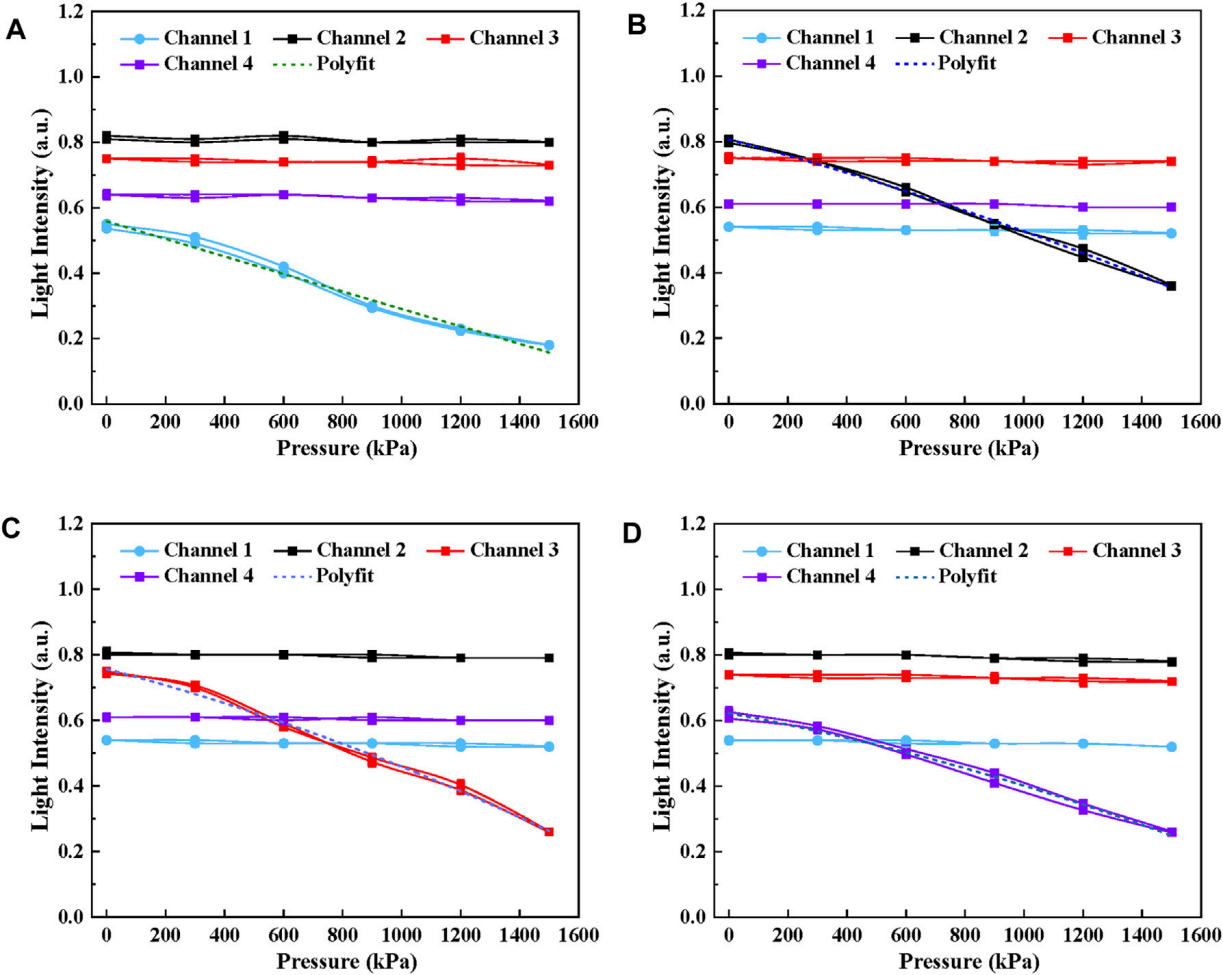
The relationship between output light intensity and the pressure ranging from 0 to 1,500 kPa. **(A)** Channel 1, **(B)** Channel 2, **(C)** Channel 3, **(D)** Channel 4.

Figure 8 shows the relationship between output light intensity and the pressure ranging from 0 to 1,500 kPa of four sensing elements, including the unloading and loading process. It can be seen that the light intensity is negatively proportional to the growth of pressure for each sensing unit. Under 1,500 kPa, the output intensity of four channels decreases to 18.2, 36.1, 25.7 and 26.1%, approximately reaches 32.7% 43.9, 36.2 and 40.6% of its initial state. The standard deviation of the results is represented in Table 2 and the error is less than 1.5%. Further, in order to make a quantitative analysis on the relationship between light intensity and pressure, polynomial fitting $y=ax^{2}+bx+c$ was performed on the sampling points of each sensing element and the fitting curve has been given in Figure 8. Table 3 demonstrates the optimal parameters of *a*, *b*, *c* and *R* ^*2*^ for four sensing units, where *R* ^*2*^ is used to show the fitting degree between the polynomial regression model and observed data. *R* ^*2*^ values of each sensing element are 0.9739, 0.9739, 0.98912, and 0.9897, respectively and it indicates a better fitness. There is a good linear relation between the light intensity and the pressure since *a* converges to 0.

**TABLE 2:** The standard deviation of experimental data.

Pressure (kPa)	Channel 1 (%)	Channel 2 (%)	Channel 3 (%)	Channel 4 (%)
0	0.000	1.155	0.000	1.528
300	0.000	1.000	0.000	0.577
600	0.000	0.577	0.000	1.155
900	0.000	0.000	1.155	1.000
1,200	0.000	0.577	1.528	0.577
1,500	0.000	0.000	1.000	0.000
1,200	0.577	0.577	1.155	0.577
900	0.577	0.577	1.528	1.000
600	1.000	1.000	1.000	0.577
300	1.000	0.000	0.577	1.155
0	0.577	0.577	0.577	0.577

**TABLE 3:** Parameters of polynomial fitting for four sensing elements.

Channel	*a*	*b*	*c*	*R* ^*2*^
**1**	1.4431 × 10^−9^	−2.6941 × 10^−4^	0.5584	0.9739
**2**	−4.2007 × 10^−8^	−2.3858 × 10^−4^	0.8071	0.9946
**3**	−6.3131 × 10^−8^	−2.3449 × 10^−4^	0.7565	0.9891
**4**	−5.5194 × 10^−8^	−1.6624 × 10^−4^	0.6229	0.9897

**FIGURE 9:**
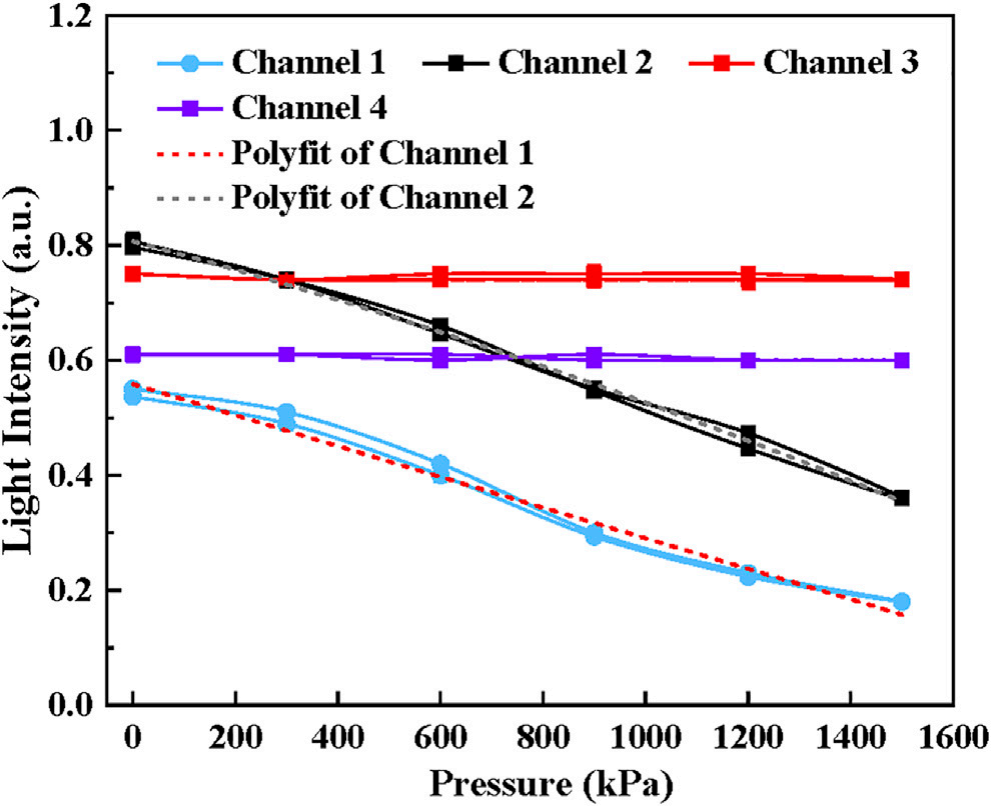
The coupling effects of the proposed sensor when tactel 1 and tactel 2 were pressurized simultaneously.

The coupling effects and crosstalk of the proposed sensor were analyzed when tactel 1 and tactel 2 were pressurized simultaneously. The result is shown in Figure 9. By comparing the parameters of polynomial fitting with Table 3, it can be concluded that the slopes for channel 1 and channel 2 remain unchanged when both channels were pressurized separately and simultaneously. Therefore, the result shows that these sensing units are independent and do not affect each other, which is consistent with the results in the simulation.

#### Hysteresis

It is noticeable that the output intensity of the sensor during loading and unloading is inconsistent, and the misalignment is usually described as hysteresis. The softness of the sensor’s material is responsible for the hysteresis because it takes a certain time to revert to its undeformed shape. The hysteresis characteristic of the sensor was analyzed by Eqs 3, 4:

$$\xi H=\frac{\Delta YL_{\max}}{y_{FS}}\times 100\%$$ (3)

$$\Delta Y_{\max}={\left|\bar{y_{uni.i}}-\bar{y_{loa.i}}\right|}_{\max}$$ (4)

Where $\;\bar{y_{unl.i}}$ is the average value of output light intensity during unloading; $\bar{y_{loa.i}}$ is the average value of output light intensity during loading, $y_{FS}$ is the full range output. The calculated results indicate that hysteresis errors for four sensing units are 2.63, 2.16, 1.22, and 1.19%.

#### Resolution

**FIGURE 10:**
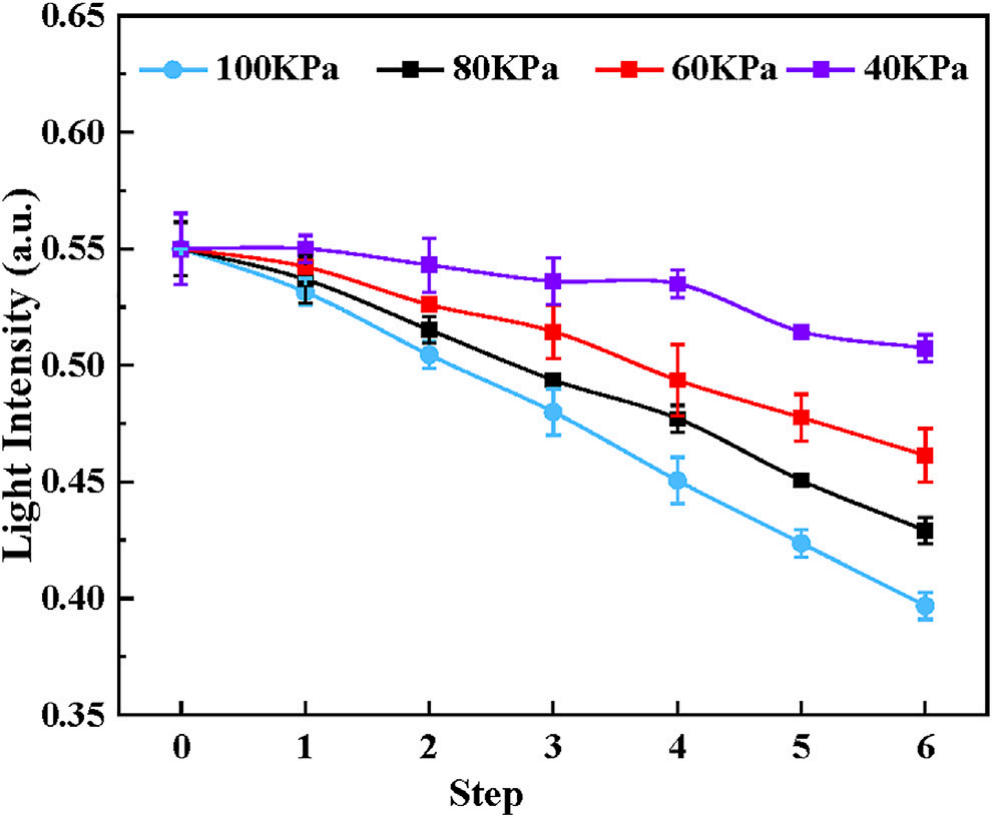
The light intensity of tactel 1 with the pressure step ranging from 40 to 100 kPa.

In this study, the resolution of the sensor represents the minimum pressure that can lead to the change of the output light intensity of the sensor. The light intensity of tactel 1 was measured with the pressure step ranging from 40 to 100 kPa. Six steps were performed at each pressure step, and each experiment was repeated five times. The experimental results are shown in Figure 10. The light intensity and pressure show a good linear relationship when the pressure steps are 100 kPa, 80 kPa, and 60 kPa. However, when the pressure step decreases to 40 kPa, the light intensity change is not apparent. So the resolution of the proposed sensor is 60 kPa (0.026 N), which is similar to tactel 2 to 4. The high resolution is of great importance to the safety of MIS.

#### Repeatability

Repeatability refers to the degree of dispersion between the measured output light intensity when the applied pressure reaches the point from the same direction and it could be calculated by Eq. 5:

$$e_{R}=\sigma_{\max}\times 100\%$$ (5)

Where $\sigma_{max}$ is the maximum value of standard deviation during loading and unloading. According to Bessel Eqs. 6, 7, $\sigma_{max}$ can be efficiently calculated.

$$\sigma_{unl_{i}}=\sqrt{\frac{1}{n-1}\sum_{j=1}^{n}{\left(y_{unl_{ij}}-\bar{y_{unli}}\right)}^{2}}$$ (6)

$$\sigma_{loa_{i}}=\sqrt{\frac{1}{n-1}\sum_{j=1}^{n}{\left(y_{loa_{ij}}-\bar{y_{loai}}\right)}^{2}}$$ (7)

*Where n* is the number of trials, in this study, *n* = 5, $\bar{y_{unli}}$ and $\bar{y_{loai}}$ are the average output intensity of unloading and loading. The repeatability for four sensing units are 1.01, 1.154, 1.527 and 1.526%, respectively.

#### Dynamic Response

To perform a dynamic calibration, the amplitude and phase response of the sensor under a given range of frequencies should be scanned. For the dynamic test, the response frequency is the inverse of the average time between consecutive peaks.

**FIGURE 11:**
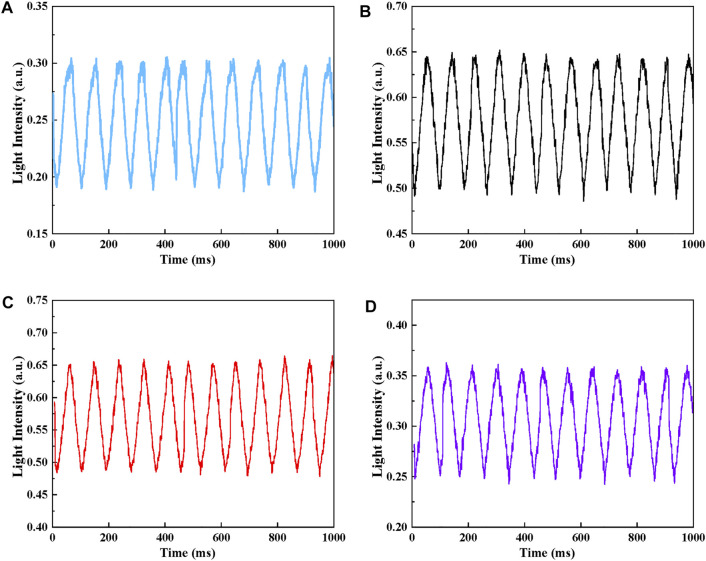
Dynamic response with a frequency of 12.5 Hz **(A)** Channel 1, **(B)** Channel 2, **(C)** Channel 3, **(D)** Channel 4.

Arduino was used to control the movement of the linear guide with a frequency of 12.5 Hz and a sawtooth-wave signal with a period of 80 ms was generated. The linear camera was used to detect the light intensity signal every 1 ms, and 1,000 points were collected continuously in 1s. The dynamic response of this proposed sensor was measured and the results are shown in Figure 11. The result shows that in the dynamic test, the fluctuation of the signal is less than 1.5%. The amplitude response was given by comparing the difference of light intensity in dynamic and static tests under the same pressure. Compared with the static test, the relative standard deviations of the light intensity for channel 1 to 4 are 1.327, 1.698,1.749 and 1.147%, respectively. As for the phase response for a given frequency, the light intensity signal is 13 ms later than the motor. So the delay time of the proposed sensor is 13 ms. Significantly, the result indicates the sensor has a good consistency and repeatability to track the dynamic signal.

## Discussion

This optical sensor shows linear response, high-resolution capacity, small hysteresis errors, high reproducibility, and fast response time. The presented results show that it is feasible to use 3D optical-based tactile sensing for multipoint force detection in MIS. Compared with the electrical-based force sensors, there is no issue of electromagnetic interference and wiring complexity. The sensor can be directly installed on the jaw to realize direct force measurement without the influence of friction and movement of the surgical instrument. Additionally, those low-cost sensors could be manufactured and produced in large batches, so their disposability and convenient installation obviate the necessity of cleaning and regularly disinfecting.

**FIGURE 12:**
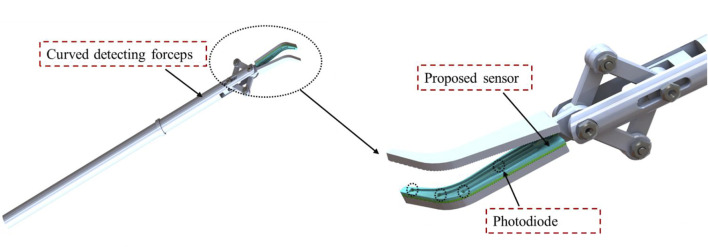
Proposed design for future work.

However, the proposed sensor is only suitable for detecting normal force in a 3D surface and the shear force was not considered. In addition, the external force is limited to the location of the tactels. In the next step, we will further miniaturize the sensor and equip it on the jaws of surgical forceps, as shown in Figure 12. The Circuit design of the detection circuit based on photodiode need to be carried out. Also, we will focus on designing the sensor capable of decoupling normal and shear force on a single tactel.

## Conclusion

This paper focuses on designing the 3D optical-based tactile sensing for detecting multiple contacts force in MIS. A sensor with four independent sensing units with the Vytaflex20 as core and Ecoflex 00-50 as cladding was proposed. The validity of the method was simulated and verified by coupling the optical and mechanical modules in COMSOL. Additionally, the static parameters of hysteresis, resolution, repeatability with the pressure range of 0–1,500 kPa were studied and the experimental results show that the static hysteresis error of the proposed sensor is less than 3%, the resolution is 0.026 N and the repeatability is less than 1.5%. The sensor’s dynamic response characteristics were also measured while the frequency was 12.5 Hz. Results show that the sensor can fully monitor the force signal in real-time, and the delay time of the proposed sensor is 13 ms. Therefore, the characteristics of small dimension, high resolution capacity, small hysteresis errors, high reproducibility and fast response time make the optical force sensor potentially useful for monitoring the force and providing accurate force feedback for MIS.

## Data Availability

The raw data supporting the conclusions of this article will be made available by the authors, without undue reservation.
